# Postpartum family planning uptake in Uganda: findings from the lot quality assurance sampling survey

**DOI:** 10.1186/s40834-023-00243-x

**Published:** 2023-08-23

**Authors:** Florence Nakaggwa, Derrick Kimuli, Kenneth Kasule, Justine Fay Katwesige, Denis Kintu, Rhobbinah Ssempebwa, Solome Sevume, Patrick Komakech, Norbert Mubiru, Baker Maggwa, Maria Augusta Carrasco, Norah Namuwenge, Rebecca N. Nsubuga, Barbara Amuron, Daraus Bukenya, Bonnie Wandera

**Affiliations:** 1 Social & Scientific Systems, Inc., a DLH Holdings Company / USAID SITES, Plot 2730 Church Road, Kironde Zone, P.O.Box 12761, Kampala, Uganda; 2 Office of Health and HIV, USAID/Uganda, US Mission Compound - South Wing, Plot 1577 Ggaba Road, P. O. Box 7856, Nsambya, Kampala, Uganda; 3 Office of Family Planning and Reproductive Health, USAID, 05.4.1A, 500 D Street SW, 20547 Washington, DC USA

**Keywords:** Postpartum, Family planning, Uganda

## Abstract

**Background:**

The initiation and use of family planning (FP) services within the first 12 months following childbirth, postpartum family planning (PPFP), promotes safe motherhood by reducing unintended pregnancies and ensuring appropriate pregnancy spacing. However, there is a paucity of information on PPFP uptake from community surveys. This study aimed to quantify the reported use of PPFP and identify predictors and barriers to PPFP uptake from a large community survey.

**Methods:**

We analysed data collected from the 2021 Lot Quality Assurance Sampling (LQAS) survey, a cross-sectional community and household survey that covered 68 districts in Uganda. The survey uses small sample sizes to designate health or administrative geographical areas which are assessed to determine whether they achieved the pre-determined target for defined indicators of interest. We abstracted and analysed data collected from mothers of children aged 12 months or younger on reproductive health and FP. PPFP use was defined as the reported use of modern FP by the mother or their partner. Associations were measured using Pearson’s chi-square test at 5% significance. Multivariate logistic regression was performed for variables that were significantly associated with PPFP use to identify the predictors of PPFP.

**Results:**

Overall, 8103 mothers of children aged less than 12 years were included in the analysis; the majority of mothers, 55.8% (4521/8103) were above 24 years while 11.7% (950/8103) were 19 years and under. 98% (7942/8103) of the mothers attended at least one antenatal care (ANC) visit and 86.3% (6997/8103) delivered at a health facility. Only 10% (814/8103) of mothers who participated in the survey reported PPFP use at the time of the survey. Reporting of PPFP use was 5 times higher among mothers of children aged 7–12 months (AOR 4.9; 95%CI 4.1–5.8), 50% higher among mothers with secondary education (AOR 1.5; 95%CI 1.0-2.3), 80% higher among breastfeeding mothers (AOR 1.8; 95%CI 1.3–2.4) and 30% lower among those that didn’t receive a health worker visit within 3 months preceding the survey (AOR 0.7; 95% CI 0.5–0.8). Among 4.6% (372/8103) who stated a reason for non-use of PPFP, the most cited reasons for not using were breastfeeding 43% (161/372), fear of side effects 26.9% (100/372), respondent/partner opposition 17.6% (48/372) and infrequent sex 12.1% (48/372).

**Conclusion:**

The analysis showed a low proportion of PPFP uptake among mothers of children under 12 years. Possible barriers included child age, education, a health worker visit, and side effects and perceived benefits of possibly improperly implementing lactation amenorrhea method. Integration of social, community and health services could provide a more holistic approach to improving PPFP uptake.

## Background

Family Planning (FP) is an essential component of health care provided during the antenatal period, immediately after delivery and during the first year postpartum [1]. The World Health Organization (WHO) recommends modern FP use immediately after birth or within 6 months for women who qualify under lactation amenorrhea (LAM) [2]. Using a modern FP method during the postpartum period is not only effective for pregnancy planning and child spacing [3] but also improves maternal and child health outcomes [4]. Closely spaced pregnancies, especially within the first year postpartum, increase the risk of preterm births, low birth weight and maternal complications [5]. Among other approaches, health services provided during antenatal care (ANC), maternity, postnatal care (PNC) and childcare are an opportunity to provide postpartum family planning services (PPFP). PPFP helps mothers choose, initiate, and continue the use of their preferred FP method for 2 years or longer, depending on the reproductive intentions of the woman or couple [2]. In as much as the postpartum fecundity date varies by woman for various reasons [6, 7], the planning of PPFP is critical in preventing unplanned pregnancies, especially in the first year after birth [8].

Across developing regions, PPFP use differs with the least rates observed in West Africa 36.3% and 39.5% in East Africa [9]. The low uptake of PPFP is attributed to various factors ranging from individual, social and health services-related challenges varying across regions and countries [10–17]. In Uganda, however, current estimates show that nationally, 35% of women use modern FP [18] moreover, only 28% of women use modern FP postpartum [17] District-level studies in Ethiopia estimate PPFP use to be 10.3% however, country-level estimates that used Demographic and Health Survey data placed Ethiopia’s PPFP use at about 29% [19]. On the otherhand, the pooled PPFP use in developing regions is estimated to be 41.2% [9] with as low as 25.5% in Ghana [9]. To address the low uptake of PPFP, it is important to understand access, use, barriers, and challenges to instituting interventions to meet postpartum mothers’ FP needs. The majority of births among young women occur in sub-Saharan Africa [1]. In Uganda, one in four births is among women in their adolescent years [18] half of whom will have a second child within their adolescent years [20]. Moreover, one study reported that only 28% of Ugandan women in the postpartum period reported PPFP use [17], however, the estimates were based on a secondary analysis of the 2011 Uganda Demographic and Health Survey (UDHS) [21] which may not reflect the current situation. Yet another constrait of the study is that it examined PPFP use among women who had a birth within 5 years preceding the UDHS. Therefore, current estimates of PPFP use are needed. The present study leverages the recently collected Lot Quality Assurance Sampling (LQAS) community survey data [22] to provide a more concise estimates of current PPFP use among mothers of children less than 12 months and possible reasons for PPFP non-use.

## Methods

### Study design and dataset used

This study was a secondary data analysis using data collected from the annual LQAS survey for 2021. LQAS surveys are done annually to assess coverage and quality of selected public health programs at subnational levels using small samples [22]. The details of LQAS activities in Uganda are described elsewhere [23, 24]. The study utilized data from the LQAS conducted from February to September 2021, which covered 64 of the then 136 administrative districts in Uganda. This study used the questionnaire issued to biological mothers of children aged 12 months or less; this data was abstracted for this analysis. The study considered 12 months to allow the examination of the predictors of PPFP in the extended post-partum period. The analysis clustered the districts according to similarities in coverage of the current implementation of FP programs.

### Sampling

Multi-stage sampling approaches were used; for each district, sub-counties were grouped into 4–6 supervision areas (SA), from each supervision area, 19–24 households depending on the number of SA were randomly selected using sampling proportional to size calculations. Using a household list at the village level, sampled households were visited, and eligible respondents were interviewed following oral consent. If there was more than one eligible person in the household, one respondent was randomly selected. Mothers of children aged 12 months or less at the time of the survey were sampled and interviewed to allow for analysis of PPFP use in the extended post-partum period.

### Study variables and measurements

The analysis outcome (dependent) variable was the current PPFP use defined as the reported use of a modern FP method at the time of the survey, categorized as a binary outcome (1 – Yes, 0 – No). The modern FP methods assessed included: Long term methods (Female sterilization and Male sterilization) and short term methods (Pill, IUD, Injectibles, Implants, Male condom, Female condom, Lactation Amenorrhea Method, Emergency contraceptives). The independent variables included: the child’s sex, child’s age, mother’s age, mother’s marital status, mother’s highest level of education, residence, pregnancy wanted, Health worker visit, ANC attendance, the gestation month at the first ANC visit, delivery place, delivery attendant and being a Member of a mother care group.

### Statistical analysis

In the descriptive analysis, the analysis computed frequencies and percentages for categorical data. Data were compared for differences in reported PPFP use using the Chi-squared test. In multivariate analysis, logistic regression analysis controlling for the effect of location for all statistically significant variables at the bivariate analysis to compute both the unadjusted odds ratio (OR) and the adjusted odds ratio (aOR) at corresponding 95% confidence intervals was conducted. Variables with p < 0.05 were considered statistically significant; the analysis was conducted in STATA version 17. Variables that were found to have a significant association with an increased likelihood of PPFP use in the univariate analysis were included in the multivariate analysis. Duration of pregnancy at 1st ANC visit was found to have a perfect correlation with the outcome variable, and therefore it was not included in the multivariable analysis to avoid issues with multicollinearity and overfitting.

## Results

### Analysis profile

**Fig. 1:**
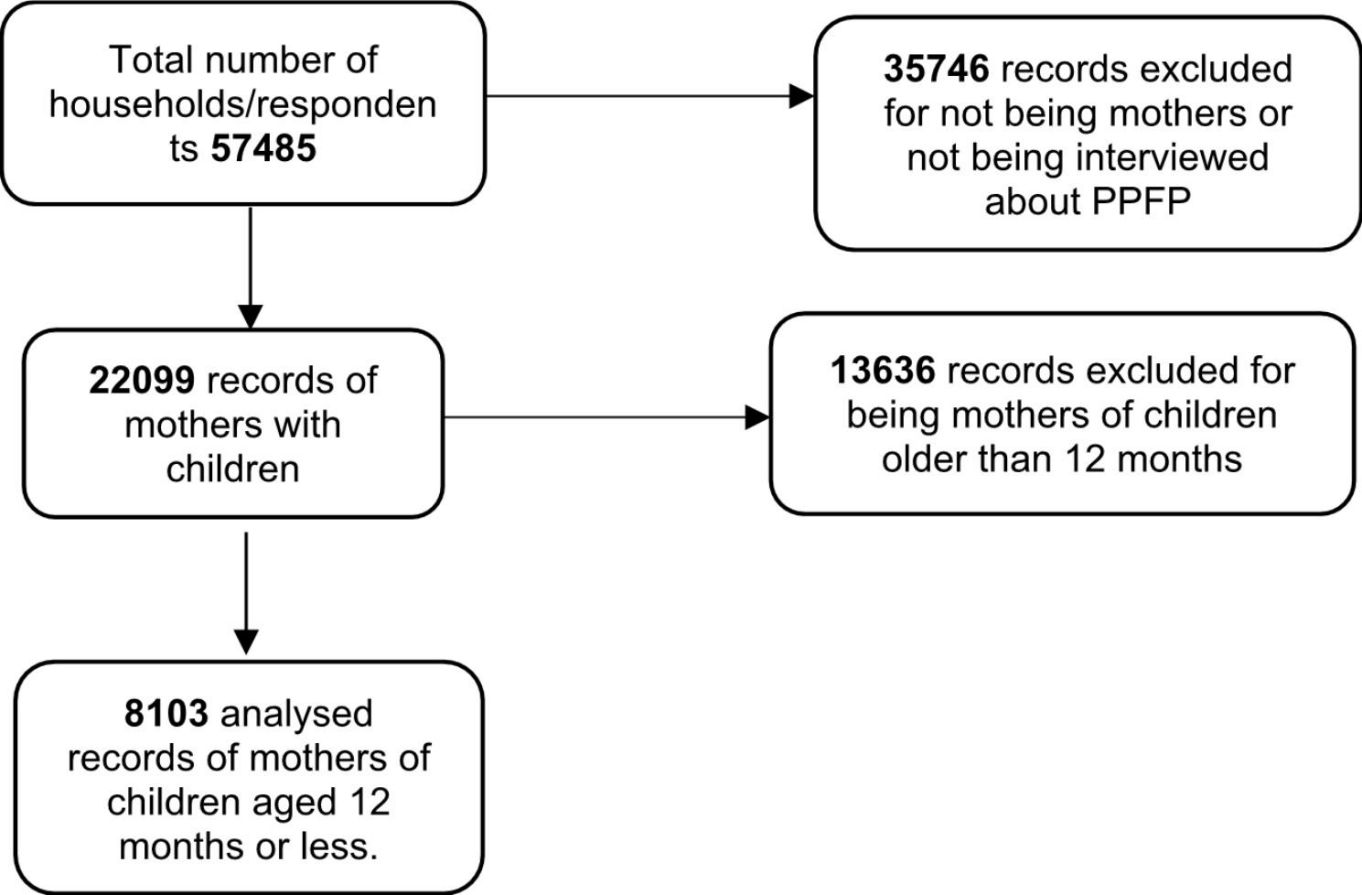
Analysis profile for the study

Overall, the LQAS 2021 survey dataset contained records of 57,485 participants. Of these, 49,382 (85%) records were excluded for the following reasons; 35,746 were not mothers or were not interviewed about PPFP, and 13,636 were mothers of children older than 11 months. Figure 1 below shows the analysis profile for the study.

### General characteristics

Table 1 shows the general characteristics of the study participants, the prevalence of PPFP and the bivariate analysis. A majority of the mothers (55.8%) interviewed were 25 years or older; 2,632 (31.5%) were 20–24 years and 950 (11.7%) were 19 years old or younger. A large proportion of the mothers (93.8%) were married or in a union, 6383 (78.8%) were rural residents and most mothers (66.9%) had primary education as their highest level of education. Slightly more than half (51.4%) of the children were female, 4632 (57.2%) were aged 0–6 months while the remaining 3471 (42.8%) were 7–12 months old. Most of the mothers (7780; 96%) were breastfeeding at the time of the survey and 7617 (94%) were members of a mother care group.

Regarding participants’ reproductive health characteristics, almost all mothers (98%) had attended ANC with a majority (29.5%) starting ANC at 3 months of pregnancy. Only 635 (8.1%) attended the recommended 8 or more visits; 7010 (86.5%) mothers delivered under skilled personnel, 671 (8.3%) delivered under non-skilled personnel and 422 (5.1%) delivered with the help of a traditional birth attendant (TBA); 6997 (86.3%) deliveries took place at a health facility and about 1051 (13%) were home deliveries.

### Prevalence of PPFP and bivariate analysis of differences in PPFP use

Of the 8103 mothers interviewed, 814 (10%) were using a modern FP method. The proportion of modern FP users was considerably higher among mothers of children aged 7–12 months (17.4%) compared to those aged 0–6 months (4.5%) and among urban residents (18.0%) compared to rural residents (7.9%). The proportion of users increased with the participant’s increasing levels of education.

A higher proportion of modern FP users was reported among mothers who attended ANC (10.1%) compared to those who did not attend (6.2%), mothers who delivered in a health facility also reported a higher FP use (10.6%) compared to those who delivered at home (6.6%) or another place (7.3%). Similarly, mothers who delivered under skilled health workers (10.6%) reported a higher FP use compared to mothers who delivered under non-skilled health workers (6.0%) or TBAs (7.1%). Mothers who indicated that their last pregnancy was wanted (10.3%) reported a slightly higher FP use compared to those whose pregnancy was unwanted (9.8%).

The unadjusted analysis showed that the age of the child (p < 0.001), the age of the mother (p = 0.004), the mother’s level of education (p < 0.001), residence (p < 0.001), months at first Antenatal Care visit (p = 0.038), place of delivery (p < 0.001) and delivery assistant (p < 0.001) were associated with a higher likelihood of PPFP use.

**Table 1:** General characteristics and bivariate analysis of differences in PPFP use

Characteristic	N (%); N = 8,103		Modern FP use		p-value
			No (n = 7289)	Yes (n = 814)	
Child sex					0.847
Male Female	3936 (48.6%) 4167 (51.4%)		3538 (89.9%) 3751 (90.0%)	398 (10.1%) 416 (10.0%)	
Child age					< 0.001*
0–6 months 7–12 months	4632 (57.2%) 3471 (42.8%)		4422 (95.5%) 2867 (82.6%)	210 (4.5%) 604 (17.4%)	
Age					0.004*
<=19 20–24 25+	950 (11.7%) 2632 (31.5%) 4521 (55.8%)		883 (92.9) 2363 (89.8%) 4043 (89.4%)	67 (7.1%) 269 (10.2%) 478 (10.6%)	
Marital status					0.255
Not in union In union	502 (6.2%) 7601 (93.8%)		459 (91.4%) 6830 (89.9%)	43 (8.6%) 771 (10.1%)	
Level of education					< 0.001*
None Primary Secondary Higher than secondary	491 (6.1%) 5424 (66.9%) 1615 (19.9%) 537 (7.1%)		448 (91.2%) 4942 (91.1%) 1405 (87.0%) 494 (86.2%)	43 (8.8%) 482 (8.9%) 210 (13.0%) 79 (13.8%)	
Residence					< 0.001*
Urban Rural	1720 (21.2%) 6383 (78.8)		1411 (82.0%) 5878 (92.1%)	309 (18.0%) 505 (7.9%)	
Currently breastfeeding					< 0.001*
Yes No	7780 (96.0%) 323 (4.0%)		7033 (90.4%) 256 (79.3%)	747 (9.6%) 67 (20.7%)	
Member of a mother care grp					0.898
Yes No	484 (6.0%) 7617 (94.0%)		438 (90.1) 6851 (89.9%)	48 (9.9%) 766 (10.1%)	
**Reproductive health characteristics**					
Attended ANC					0.102
Yes No	7942 (98%) 161 (2%)		7138 (89.9%) 151 (93.8%)	804 (10.1%) 10 (6.2%)	
Months at 1st ANC visit					0.038*
1 2 3 4 5	511 (6.4%) 1132 (14.3%) 2347 (29.5%) 1991 (25.1%) 1961 (24.7%)		472 (92.4%) 1005 (88.8%) 2111 (89.9%) 1767 (88.8%) 1783 (90.9%)	39 (7.6%) 127 (11.2%) 236 (10.1%) 224 (11.3%) 178 (9.1%)	
No. of visits					0.481
1–7 8+	7207 (91.9%) 635 (8.1%)		6474 (89.8%) 576 (90.7%)	733 (10.2%) 59 (9.3%)	
Delivery place					< 0.001*
Health facility Home Other place	6997 (86.3%) 1051 (13.0%) 55 (0.7%)		6256 (89.4%) 982 (93.4%) 51 (92.7%)	741 (10.6%) 69 (6.6%) 4 (7.3%)	
Delivery person					< 0.001*
Skilled Non skilled TBA	7010 (86.5%) 671 (8.3%) 422 (5.1%)		6266 (89.4%) 631 (94.0%) 392 (92.9%)	744 (10.6%) 40 (6.0%) 30 (7.1%)	
Last pregnancy wanted					0.493
Yes No	5285 (67.0%) 2728 (34.0%)		4740 (89.7%) 2460 (90.2%)	545 (10.3%) 268 (9.8%)	

### Predictors of PPFP use

The multivariate analysis showed that mothers of children aged 7–12 months were 5 times more likely to report modern FP use compared to mothers of younger children (AOR 4.9; 95%CI 4.1–5.8). As the mother’s level of education increased, the likelihood of using a modern FP method postpartum also increased, corresponding to increases of 30% (AOR 1.3; 95%CI 0.9–1.9), 50% (AOR 1.5; 95%CI 1.0-2.3) and 60% (AOR 1.6; 95%CI 1.0-2.5) among mothers with primary, secondary and higher than secondary education respectively compared to those mothers with no education. Furthermore, the odds of modern FP use were 20% higher among rural mothers (AOR 1.2; 95%CI 0.9–1.6) compared to urban mothers, and 80% higher among non-breastfeeding mothers (AOR 1.8 95%CI 1.3–2.4) compared to those that were breastfeeding at the time of the interview (Table 2).

**Table 2:** Multivariate analysis of factors associated with PPFP Use

Characteristic	Unadjusted OR (95%CI)	P-value	Adjusted OR (95%CI)	p-value
Child age				
0–6 months	1 (Reference)		1 (Reference)	
7–12 months	4.4 (3.8–5.2)	< 0.001*	4.9 (4.1–5.8)	< 0.001*
Mother’s Age				
25+	1 (Reference)		1 (Reference)	
20–24 <=19	1.0 (0.8–1.1) 0.6 (0.5–0.8)	0.638 0.001*	1.0 (0.9–1.2) 0.8 (0.6-1.0)	0.697 0.082
Level of education				
None	1 (Reference)		1 (Reference)	
Primary Secondary Higher than secondary	1.0 (0.7–1.4) 1.6 (1.1–2.2) 1.7 (1.1–2.5)	0.923 0.012* 0.011*	1.3 (0.9–1.9) 1.5 (1.0-2.3) 1.6 (1.0-2.5)	0.140 0.037* 0.059
Residence				
Urban	1 (Reference)		1 (Reference)	
Rural	0.4 (0.3–0.5)	< 0.001*	1.2 (0.9–1.6)	0.124
Currently breastfeeding				
Yes	1 (Reference)		1.8 (1.3–2.4)	
No	2.5 (1.9–3.3)	< 0.001*	1.8 (1.3–2.4)	0.001*
Delivery place				
Health facility	1 (Reference)		1.0 (0.6–1.7) 0.9 (0.3-3.0)	
Home Other places	0.6 (0.5–0.8) 0.7 (0.2–1.8)	< 0.001* 0.429	1.0 (0.6–1.7) 0.9 (0.3-3.0)	0.984 0.876
Delivery person				
Skilled	1 (Reference)		1 (Reference)	
Non-skilled TBA	0.5 (0.4–0.7) 0.6 (0.4–0.9)	< 0.001* 0.023*	0.8 (0.5–1.5) 0.9 (0.5–1.6)	0.522 0.659
Health worker visit				
Yes	1 (Reference)		0.7 (0.5–0.8)	
No	0.6 (0.5–0.7)	< 0.001*	0.7 (0.5–0.8)	0.001*

### Reasons for non-PPFP use

**Fig. 2:**
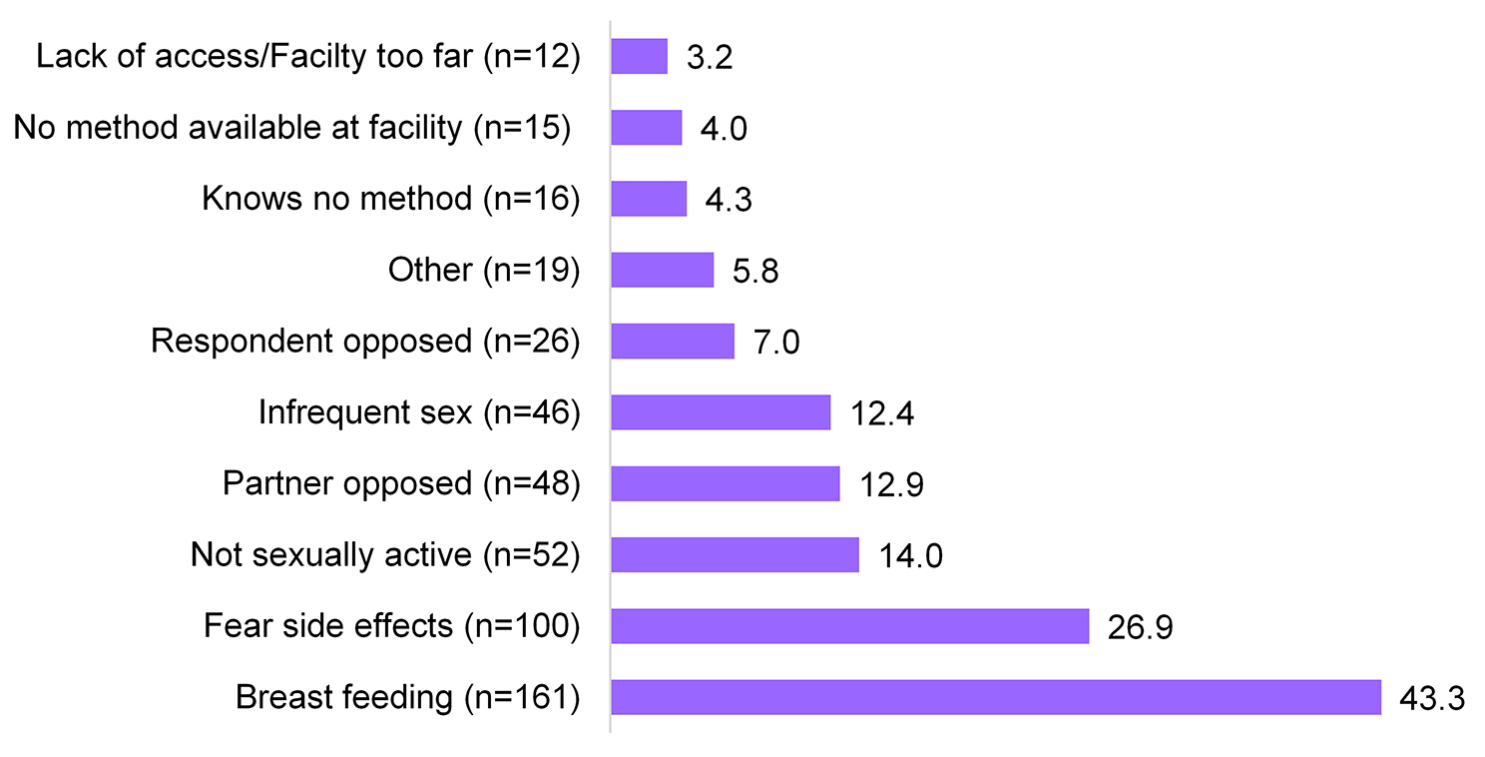
Reasons for non-use of PPFP, percentages are shown

Figure 2 shows the reasons given for non-PPFP use. Of the 7,289 (90%) women who reported not using modern FP in the post-partum period, 372 (4.6%) cited reasons for non-use. The most cited reason was breastfeeding 43.3% (161/372) followed by fear of side effects 26.9%(100/372) while the least cited reasons were accessibility to the facility and lack of FP methods at the facility [3.2% (12/372) and 4.0% (15/372) respectively].

## Discussion

The study findings showed that only one in ten mothers of children aged 12 months or less, i.e., postpartum mothers, in the surveyed regions reported the current use of PPFP. A smaller sample and localized survey in Northwest Ethiopia, a country of similar social and cultural context to Uganda, found similar results (10.3%) [19]. The proportion reported by the present study is much lower than that observed by a similar study of a secondary analysis of the 2011 UDHS dataset at 3 in 10 [17]. However, the latter study used wider inclusion criteria (i.e. women who had given birth within 5 years preceding the study). This finding suggests that the prevalence of early PPFP in Uganda may be much lower than what is estimated or routinely used for planning. Studies have linked this low PPFP use to postpartum women underscoring the risk of pregnancy during breastfeeding [25]. As a result, the significance of PPFP becomes evident as an indispensable component within the spectrum of maternal and newborn health care services [2, 26]. Therefore, the observed low PPFP uptake could mean some underlying challenges with the integration of PPFP services within the continuum of care in Uganda that need to be examined further. Short of this, the country may continue to struggle with closely timed births within the population [20] which are associated with higher maternal and infant mortality rates, some of which could, in part, be averted through scaling up of PPFP [5].

Mothers of older children were more likely to use PPFP than mothers of younger children. This could be one of the reasons why a higher uptake of PPFP was observed in the secondary analysis of the 2011 UDHS dataset which considered mothers of children up to 5 years [17]. The resumption of sexual activity after delivery varies based on many factors, including the type of delivery for which the healing period can last up to 6 months [27, 28]. However, this may also imply a delay in PPFP uptake, therefore, the findings suggest that mothers in Uganda are likely to delay the use of PPFP to a much later period than recommended. On the other hand, the delay and prevalence may also be attributed to the generally low attendance at PNC services, which is only modest within the first few days of delivery in the country [29, 30]. This increases the likelihood of unplanned pregnancies that may complicate other maternal and child health outcomes [8]. Moreover, previous work in low-income countries has also shown that despite a substantially expressed need to delay or prevent pregnancy during the postpartum period, PPFP is generally low [31]. The study findings of the present study also suggest that an increase in education increased PPFP uptake, a finding that has been supported by similar studies in Uganda [12, 17], Ethiopia [11, 19, 32] and beyond [10]. Educated women are more likely to be aware of the benefits of PPFP, have access to information about PPFP, and have resources needed such as money and transportation or more need for PPPF since they may be working [10, 17]. The association between education and PPFP use highlights the importance of addressing educational disparities to improve PPFP uptake among mothers.

The utilization of PPFP was found to be lower among mothers who had not received a health or social worker visit within three months before the survey. In the context of primary health care, community workers play a vital role in expanding access to health services, although their utilization remains underutilized despite proven benefits [33]. Engaging community workers can effectively increase the availability and uptake of FP services by implementing community-based dispensing and education approaches, thereby bringing services directly to the people rather than requiring individuals to seek them out. Zambia has demonstrated the positive impact of such interventions on decision-making regarding childbearing [34]. Conversely, the present study observed higher odds of PPFP use among mothers who were not breastfeeding which may be attributed to several factors. It is possible that breastfeeding mothers were aware of the benefits of the LAM but had inadequate knowledge or did not fully adhere to the criteria for LAM use [35, 36]. Additionally, it is worth noting that only a small percentage (5%) of mothers who reported using PPFP cited LAM as their preferred method [2, 36, 37]. However, it is important to highlight that a significant majority (43%) of mothers who did not use PPFP mentioned breastfeeding as the reason for non-utilization. Although this study did not establish the specific underlying reason for this observation, such as whether mothers believed that breastfeeding provided sufficient protection against pregnancy or if they were aware of contraindications, it could be worth exploring in future surveys. Similar to other medical interventions [38–41], modern FP methods face challenges in addressing concerns related to side effects [42–45]. In Uganda, FP side effects have been identified as the primary reason for discontinuation of FP use [46]. This study also revealed that the fear of side effects ranked as the second most common reason for not using PPFP marking this concern as one of the major obstacles to PPFP use in Uganda.

This study is one of few studies that have examined PPFP using the most routine survey data district-based survey in Uganda that is often not used for such analysis. It presents important insights into the prevailing situation regarding PPFP in the country since LQAS data provides routine and reliable district, regional and national estimates. Its strengths include a large sample size that allowed for representation from different districts and regions improving its generalizability. Moreover, this study considers women who have given birth within a year preceding the survey. This provides for a better estimate of PPFP within a more critical time, i.e. within the first year of birth of a child. PPFP is essential for enabling mothers to adequately space their children and limit untimed pregnancies, which are both linked to maternal and child wellbeing [5]. The study’s population also allowed for better accuracy of recalled information, given that it was among mothers who had given birth within a year of the survey, unlike 1 a similar study in the country that considered mothers of children up to 5 years [17]. However, the study being a secondary analysis did not control for other factors that could influence PPFP uptake that were not collected during the survey, making it challenging to establish a direct causal relationship between the analyzed factors and the observed outcome. However, this study considered all relevant variables available in the survey data based on literature evidence and may highlight the need for additional variables to be included in future LQAS surveys.

## Conclusions and implications

The findings of this study underscore the challenges faced in the utilization of PPFP among mothers of children aged 12 months or less. The low rate of PPFP use, reported by only one in ten mothers in the surveyed regions, reflects a pressing need to enhance access to and utilization of effective FP methods during the postpartum period. The study findings highlight the broader implications of the observed low PPFP uptake in terms of closely timed births and associated maternal and infant health risks. To address these challenges, early postpartum uptake of FP and incorporation of comprehensive information and counselling on PPFP during antenatal and postnatal health education is crucial. Additionally, efforts should be directed towards addressing concerns related to side effects, as they emerged significantly as a barrier to PPFP use. Engaging and empowering community workers can play a vital role in expanding access to PPFP by implementing community-based interventions that directly reach postpartum mothers. Such approaches have shown promise in improving decision-making regarding FP in other contexts. By integrating these strategies and addressing the identified predictors and barriers, we can enhance PPFP utilization, promote maternal and infant health, and empower women to make informed choices about their reproductive well-being.

## Data Availability

The dataset used for this study is available upon request from the corresponding author at Social & Scientific Systems, Inc., (SSS), a DLH Holdings Company.
